# Integrating 16S rRNA Gene Sequencing and Metabolomics Analysis to Reveal the Mechanism of L-Proline in Preventing Autism-like Behavior in Mice

**DOI:** 10.3390/nu17020247

**Published:** 2025-01-10

**Authors:** Jingjing Fang, Seong-Gook Kang, Kunlun Huang, Tao Tong

**Affiliations:** 1 Key Laboratory of Precision Nutrition and Food Quality, Key Laboratory of Functional Dairy, Ministry of Education, College of Food Science and Nutritional Engineering, China Agricultural University, Beijing 100083, China; 2 Department of Food Engineering and Solar Salt Research Center, Mokpo National University, Muangun 58554, Republic of Korea; 3 Key Laboratory of Safety Assessment of Genetically Modified Organism (Food Safety), Ministry of Agriculture, Beijing 100083, China; 4 Beijing Laboratory for Food Quality and Safety, Beijing 100083, China

**Keywords:** autism spectrum disorder, gut microbiota, gut metabolites, L-proline

## Abstract

Background/Objectives: Autism spectrum disorder (ASD) is characterized by impaired social interaction and repetitive stereotyped behavior. Effective interventions for the core autistic symptoms are currently limited. Methods: This study employed a valproic acid (VPA)-induced mouse model of ASD to assess the preventative effects of L-proline supplementation on ASD-like behaviors. The method of 16S rRNA sequencing and untargeted metabolomics analyses were conducted to investigate the modulation of gut microbiota and gut metabolites by L-proline. Results: The results indicated that L-proline supplementation significantly prevented ASD-like behavioral disorders, including alleviating social communication deficits and reducing repetitive behavior in the ASD mice. The 16S rRNA sequencing analysis revealed that L-proline regulated the composition and structure of gut microbiota. L-Proline supplementation enhances the abundance of the *Verrucomicrobia* at the phylum level and the *Akkermansia* at the genus level, while concurrently reducing the abundance of the *Patescibacteria* at the phylum level, as well as the *Ileibacterium*, *Candidatus_Saccharimonas*, and *Lachnospiraceae_UCG-006* at the genus level in the VPA-induced mouse model for ASD. Additionally, the untargeted metabolomics results indicated that L-proline also modified the gut metabolite profiles. Functional analysis of the gut microbiota and KEGG pathway enrichment analysis of differential metabolites between the L-proline-supplemented and VPA groups corroborated that L-proline decreased pathways related to nucleotide metabolism, taurine and hypotaurine metabolism, and pyruvate metabolism, while increasing pathways involved in alpha-linolenic acid metabolism and phenylalanine, tyrosine, and tryptophan biosynthesis. The integrative metabolomic and microbiome analyses showed strong connections between the gut metabolites and gut microbiota affected by L-proline. These findings suggest that the modulatory effects of L-proline on gut microbiota and its metabolites may play a crucial role in preventing autism in mice. Conclusions: These findings suggest that dietary L-proline may represent a viable, effective option for preventing the physiological and behavioral deficits associated with ASD in mice.

## 1. Introduction

Autism spectrum disorders (ASD) constitute a class of neurodevelopmental impairments that encompass a range of cognitive, emotional, relational, and behavioral domains [1,2]. The etiology of ASD is intricate, and it is postulated that ASD emerges from the interplay between genetic and environmental factors [3,4]. To date, identifying a common causative factor that could account for a substantial percentage of ASD cases remains an unresolved issue within the field. ASD affects approximately 1% of the global population collectively and is primarily characterized by impairments in social interaction and the exhibition of repetitive behaviors [5]. Despite evidence suggesting that early behavioral and educational interventions can alleviate the symptoms of ASD patients, the availability of effective pharmacological treatments remains limited. Risperidone and aripiprazole are the sole treatments approved by the U.S. Food and Drug Administration for managing irritability in pediatric patients with ASD, necessitating the development of additional therapeutic interventions [6].

Recent studies have increasingly directed their focus toward investigating the potential association between gut microbiota and neurodevelopmental outcomes, specifically in the context of ASD [7]. Emerging evidence indicates a potential linkage between alterations in the gut microbiome of autistic individuals and the core symptoms of the condition, thereby highlighting the pivotal role of gut dysbiosis in the initiation and progression of ASD [8]. Alterations in the microbiome frequently lead to modifications in metabolic profiles, subsequently influencing the availability and diversity of nutrients as well as microbial metabolites [9]. These rodent models, specifically those exhibiting ASD-like phenotypes following prenatal exposure to valproic acid (VPA), demonstrate not only behavioral characteristics that are indicative of ASD but perturbations in the gut microbiota and associated metabolite profiles [10]. Accordingly, these models serve as a robust platform for investigating autistic behaviors, elucidating the underlying molecular mechanisms, and assessing the efficacy of potential therapeutic interventions [11].

Notably, our previous unpublished data have confirmed a decrease in L-proline among the gut metabolites of individuals with ASD. Furthermore, studies have reported significantly lower serum or plasma L-proline levels in ASD patients compared to normal individuals [12,13,14], hinting at a potential association between these gut metabolites and the pathophysiology of ASD. Research has demonstrated that the administration of 5-aminovaleric acid, a product derived from the *Stickland* fermentation of L-proline, from conception through adulthood, results in a reduction of repetitive behaviors in BTBR mice [15]. Our previous research has shown that supplementing L-proline from adolescence to adulthood has therapeutic effects on autism-like behaviors in mice [16]. However, it remains unclear whether dietary proline supplementation from maternal rats during pregnancy can effectively prevent ASD-like behavioral disorders. On the other hand, studies have reported that the prenatal and early postnatal periods represent a critical window for neurodevelopment [17], and an increasing number of investigations are beginning to elucidate the significance of the maternal gut microbiome in influencing neurobehavioral outcomes in offspring [18]. Consequently, the primary prevention of ASD, which focuses on pregnancy and the early modulation of key effector molecular pathways, is anticipated to be the most efficacious intervention strategy [17].

Based on these findings, we orally administered L-proline to the VPA-induced mouse model of ASD in the PRO-VPA group, starting from conception and continuing through adulthood, to encompass both prenatal and postnatal neurodevelopmental periods. The behavioral manifestations of these mice were then compared with those of the untreated mice in the VPA group. Subsequently, 16S rRNA sequencing and an untargeted metabolomics analysis were utilized to elucidate the potential mechanisms underlying the prevention of autism-like behavioral disorders by L-proline.

## 2. Materials and Methods

### 2.1. Animals and Experimental Design

All experimental procedures were approved by the Laboratory Animal Welfare and Animal Experimental Ethical Committee of China Agricultural University (Approval Number: AW30803202-4-5, Beijing, China). Fourteen-week-old C57BL/6J female and male mice were purchased from Vital River Laboratories (Beijing, China) and housed in specific pathogen-free conditions at the Animal Centre (SYXK (Jing) 2020-0052), maintained at a relative humidity of 40–70%, a temperature of 22 ± 2 °C, and a 12-h light-dark cycle.

Adult male and female C57BL/6J mice were bred using a monogamous breeding scheme at night, with pregnancy confirmed by the presence of vaginal plugs the following morning, designated as embryonic day 0.5 (E0.5). Pregnant mice were individually housed and randomized into three groups: SAL, VPA, and PRO-VPA groups. Mice in the SAL and VPA groups, along with their offspring, were provided with sterile water, whereas those in the PRO-VPA group received sterile water supplemented with 1% (*w*/*w*) L-proline (99% purity, Sigma). On embryonic day 12.5 (E12.5), pregnant mice in the VPA and PRO-VPA groups were intraperitoneally injected (i.p.) with 600 mg/kg of VPA (98% purity, Sigma, St. Louis, MO, USA), while mice in the SAL group received an equivalent volume of 0.9% saline. Male offspring were weaned at postnatal day 21, resulting in 15 mice in the postnatal SAL group, 19 mice in the postnatal VPA group, and 11 mice in the postnatal PRO-VPA group. All animals had ad libitum access to food and water during the experimental period.

**Figure 1:**
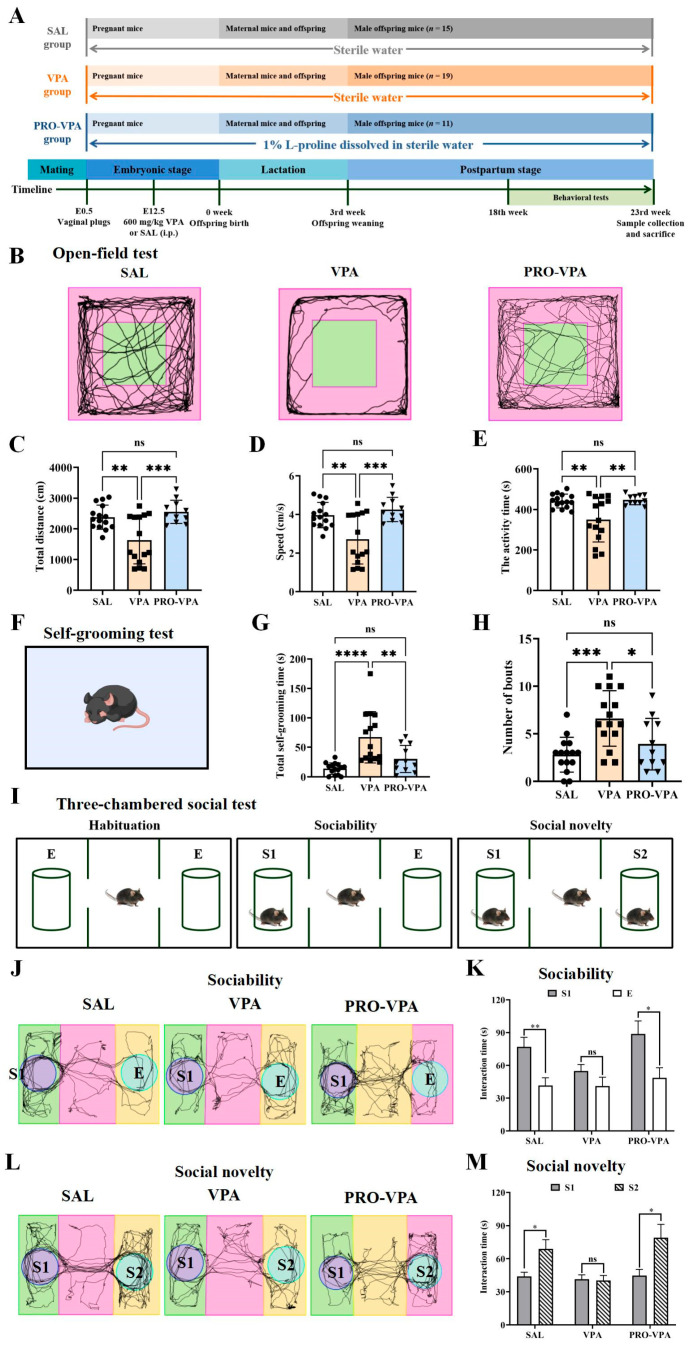
L-proline prevents autism-like behaviors in the VPA-induced ASD mouse model. (**A**) Schematic representation of the experimental procedure. (**B**) Representative trajectory diagram of the open-field test. (**C**) The total distance traveled in the open-field test. (**D**) The travel speed in the open-field test. (**E**) The activity time in the open-field test. (**F**) The diagram of the self-grooming test. (**G**) The total time of self-grooming. (**H**) The number of self-grooming bouts. (**I**) The diagram of the three-chambered social test. (**J**) Representative trajectory diagram of sociability test. (**K**) Time spent exploring strange mouse 1 (S1) or empty cage (E). (**L**) Representative trajectory diagram of social novelty test. (**M**) Time spent exploring strange mouse 1 (S1) or strange mouse 2 (S2). The bars indicate mean ± SEM values. All data were statistically analyzed using one-way ANOVA (**B**–**G**) or unpaired two-tailed Student’s *t*-test (**J**,**L**). Significance was set at ns, not significant (*p* > 0.05), * *p* < 0.05, ** *p* < 0.01, *** *p* < 0.001, and **** *p* < 0.0001. *n* = 11–19.

At 18 weeks of age, the offspring mice underwent behavioral testing in the following sequence: open-field test, self-grooming test, and three-chambered social test. Following completion of the behavioral assessments, the mice were euthanized at 23 weeks of age after a 6-h fasting period (from 8:00 a.m. to 2:00 p.m.). Euthanasia was conducted under isoflurane anesthesia via cervical dislocation in accordance with humane protocols. Subsequently, cecal content samples were collected and stored at −80 °C for subsequent analysis. Figure 1A illustrates a detailed timeline of the animal experimental procedures.

### 2.2. Behavioral Test

#### 2.2.1. Open-Field Test

During the open-field test, the mice were placed in the center of a square-shaped open-field arena (40 × 40 × 30 cm) and permitted to freely explore the environment. Prior to testing, the arena was sanitized with 75% ethanol followed by rinsing with water. Subsequently, the mice were introduced into the arena and allowed to explore for a duration of 10 min while being tracked. Locomotion was recorded using a ceiling-mounted camera interfaced with ViewPoint video tracking software (Labmaze V3.0). The collected data were analyzed to determine total travel distance, speed, and activity time by observers who were blinded to the experimental grouping.

#### 2.2.2. Self-Grooming Test

The self-grooming test was conducted as previously described [16]. Briefly, the test was administered in a clean, empty plastic cage (30 × 20 × 15 cm) for a duration of 20 min, with no bedding material provided to avoid digging behavior. Each mouse underwent a 10-min habituation period prior to the test. Subsequently, the self-grooming behavior of each mouse was recorded for an additional 10 min using a digital camera. The test was evaluated by observers who were blinded to the experimental grouping. The number of self-grooming bouts and the total cumulative time spent on self-grooming were obtained from the video recordings for each mouse.

#### 2.2.3. Three-Chambered Social Test

The three-chambered social test was conducted as previously described [16]. Firstly, the experimental mouse was habituated for the initial 10-min session in an empty plexiglass cage (60 × 40 × 25 cm) divided into three chambers. Sociability was then assessed during the subsequent 10-min session, where the mouse had the opportunity to interact with either an empty wire cage (E) or a wire cage containing an age-, genotype-, and sex-matched stranger mouse (S1). The duration of interaction was measured by the time the mouse spent sniffing or climbing on the empty cage or the cage housing S1. During the third 10-min period, a second stranger mouse (S2) was introduced into the empty wire cage to evaluate the social novelty preference of the experimental mouse. The time spent interacting with the empty cage, S1, or S2 was recorded and measured by independent, trained observers using automated ANY-Maze software (Labmaze V3.0), with the experimenters blinded to the treatment group assignment.

### 2.3. DNA Extraction and 16S rRNA Gene Sequencing

A NanoDrop 2000 spectrophotometer manufactured by Thermo Fisher Scientific (Wilmington, DE, USA) was utilized to quantify the purity and concentration of the total DNA extracted from cecal content samples. Subsequently, the 16S rRNA gene segments corresponding to the V3–V4 regions were amplified from the extracted DNA utilizing the primers 806R (5′-GGACTACHVGGGTWTCTAAT-3′) and 338F (5′-ACTCCTACGGGAGGCAGCAG-3′). Amplification was achieved through the execution of 27 cycles of polymerase chain reaction, with each cycle consisting of denaturation at 95 °C for 30 s, annealing at 55 °C for 30 s, and extension at 72 °C for 45 s. For paired-end sequencing of the amplified products, the Illumina MiSeq sequencing platform (Illumina, San Diego, CA, USA), along with PE300 reagents provided by Majorbio Biomedical Technology Co. (Shanghai, China), was employed.

The raw data were processed on the Majorbio Cloud platform (https://cloud.majorbio.com/, Shanghai, China). Following demultiplexing, the sequences were merged using FLASH (version 1.2.11) and subsequently underwent quality filtering with fastp (version 0.19.6). High-quality sequences were then denoised utilizing the DADA2 plugin within the QIIME2 pipeline (version 2020.2). The resultant sequences, termed amplicon sequence variants (ASVs), represent the conventional nomenclature for DADA2-denoised sequences. For ASV classification, the built-in Naive Bayes consensus classifier within QIIME2 was utilized, with the SILVA 16S rRNA database (version 138) serving as the reference for ASV categorization.

The α diversity indices at the ASV level were calculated using the QIIME2 software (version 2020.2). Statistical significance was set at *p* < 0.05, with false-discovery rate (FDR)-adjusted *p*-values calculated using Welch’s correction to account for potential biases. Principal coordinates analysis (PCoA) visualizations were generated on the Majorbio Cloud platform (https://cloud.majorbio.com/ accessed on 11 December 2024, Shanghai, China), followed by a weighted UniFrac distance-based analysis of similarity (ANOSIM) to assess clustering patterns. Differences in bacterial taxa, ranging from the phylum to the genus level, were evaluated using a linear discriminant analysis (LDA) coupled with an LDA effect size (LEfSe) analysis, applying two filters: *p* < 0.05 and LDA score > 4. Given the potential for high false-positive rates associated with LEfSe, as previously reported [19], an additional analysis of the composition of microbiomes (ANCOM) was conducted using the ANCOM 4.0.2 package in R to refine the identification of bacterial taxa enriched across sample groups. The significance of ANCOM was defined as W > 0.6. Finally, PICRUSt2 was employed to predict the functional capabilities of the gut microbial communities.

### 2.4. Untargeted Metabolomic Analysis

Shanghai Majorbio Bio-pharm Technology Co., Ltd. conducted an untargeted metabolomic analysis of cecal content samples. For metabolite extraction, a solution consisting of methanol and water in a volume ratio of 2:1 (*v*/*v*), containing internal standards, was employed. Each sample was supplemented with 400 μL of the extraction solvent and subsequently milled in a grinder for a duration of 6 min at −10 °C with a frequency of 50 Hz. This was followed by a low-temperature ultrasonic extraction step for 30 min at 5 °C with an ultrasonic frequency of 40 KHz. Subsequently, the samples were allowed to equilibrate at −20 °C for 30 min, followed by centrifugation at 13,000× *g* for 15 min at 4 °C. The resultant supernatant was then pipetted for further analytical procedures.

The instrumental platform utilized for the untargeted metabolomics analysis in this study was the Ultra High Performance Liquid Chromatography Tandem Fourier Transform Mass Spectrometry UHPLC-Exploris 240 system (Thermo Fisher, USA). For liquid chromatography (LC) separation, an ACQUITY UPLC HSS T3 column (100 mm × 2.1 mm, 1.8 µm; manufactured by Waters, Milford, CT, USA) was employed. Mobile phase A comprised 95% water and 5% acetonitrile (supplemented with 0.1% formic acid), while mobile phase B consisted of 47.5% acetonitrile, 47.5% isopropanol, and 5% water (also containing 0.1% formic acid). The injection volume was set to 3 μL, and the column temperature was maintained at 40 °C. The samples were ionized through electrospray, and the mass spectrometry signals were acquired in both positive (ES+) and negative (ES−) ion scanning modes.

The raw data were processed utilizing Progenesis QI v3.0 software (Waters Corporation, Milford, CT, USA). Subsequent to normalization based on total peak intensity, the processed data were uploaded and imported into the ropls R package (version 1.6.2), where they underwent multivariate data analysis. This included partial least squares discriminant analysis (PLS-DA) to investigate alterations in metabolic profiles across different groups. The Student’s *t*-test was employed to determine the *p* between pairs of metabolite groups. Metabolites were screened based on a variable importance in projection (VIP) > 1.0 and a *p* < 0.05. The Human Metabolome Database (HMDB) was utilized for annotating the identified differential metabolites. Differential metabolites between the VPA and PRO-VPA groups were imported into the Kyoto Encyclopedia of Genes and Genomes (KEGG) database for further analysis of their functions and metabolic pathways. Only pathways with a *p* < 0.05 were considered as significantly altered.

### 2.5. Statistical Analysis

The sample size of the litters was decided from previous works on the VPA model in this mouse strain and behavioral analyses [20] and all the male offspring from different litters were selected for the behavioral analyses. The determination of the number of animals for the molecular analyses was guided by the previous, similar literature, in which sample sizes of *n* = 3–5 are typically employed for the analysis of gut microbiota and metabolomes [16,21,22]. No statistical methods were used to predetermine the sample size. No outliers were considered, and all collected data were used in statistical analyses.

The data were presented as the mean ± standard error of the mean (SEM). Comparisons involving a single variable were performed using an unpaired, two-tailed Student’s *t*-test. Differences among multiple groups with a single variable were assessed using a one-way analysis of variance (ANOVA). All statistical analyses were conducted utilizing GraphPad Prism 9.5 software (GraphPad, San Diego, CA, USA). The level of significance was denoted as follows: * *p* < 0.05, ** *p* < 0.01, *** *p* < 0.001, and **** *p* < 0.0001.

## 3. Results

### 3.1. L-Proline Prevents Autism-like Behavior in the VPA-Induced ASD Mouse Model

The preventive impact of L-proline on behavioral disorders reported in the prenatal VPA-exposed male mice was investigated by assessing locomotor activity, repetitive stereotypical behavior, and social dysfunction. Initially, an open-field test was employed to evaluate the effect of L-proline supplementation on locomotor activity. In this test, the VPA-exposed mice exhibited significantly decreased total distances traveled, travel speed, and activity time (Figure 1B–E) compared to mice in the SAL control group, aligning with previous findings [23,24]. Notably, L-proline supplementation significantly prevented the abnormal reduction in total distance traveled and speed observed in the open-field test among mice with ASD characteristics (Figure 1B–E). ASD patients often display repetitive stereotypical behaviors and social impairments. Intriguingly, our results indicated that the VPA-treated mice spent significantly more time on self-grooming compared to the SAL group, an effect that was substantially prevented by L-proline supplementation (Figure 1G,H).

Given that social dysfunction is a prominent characteristic of individuals with ASD and that mice exposed to VPA exhibit impaired social behavior, a three-chamber social test was conducted to investigate the sociability and preference for social novelty of these mice. To assess sociability, the time spent interacting with the stranger mice 1 (S1) versus an empty wired cage (E) was evaluated. As anticipated, mice in the SAL control group displayed normal social behavior, evident by their preference for interacting with the stranger mice. Conversely, the VPA-treated mice did not exhibit a preference for the stranger mice over empty cages, indicating impaired sociability, which was ameliorated by L-proline supplementation (Figure 1J,K). Additionally, the VPA-treated mice failed to demonstrate a normal preference for social novelty. Dietary L-proline restored social novelty preferences in the VPA-treated mice, with the performance of mice in the PRO-VPA group being comparable to that of the SAL group (Figure 1L,M).

Consequently, these findings demonstrate that dietary L-proline significantly prevented ASD-like behavioral disorders in the VPA-induced ASD mouse model, as evidenced by improvements in social communication deficits and reductions in repetitive behavior.

### 3.2. L-Proline Modulates the Structure of Gut Microbiota in the VPA-Induced ASD Mouse Model

Recent experimental studies conducted on mice have provided evidence that gut microbiota can play a modulatory role in brain-centered disorders, including ASD [25]. Additionally, dietary L-proline has been identified as a potential regulator of both the gut microbiome and metabolome [16]. To investigate whether L-proline supplementation could alter the gut microbial composition in mice treated with VPA, we conducted an analysis of microbial communities using 16S rRNA sequencing. Initially, we assessed the impact of L-proline supplementation on the α diversity and β diversity of the gut microbiota in a VPA-induced ASD mouse model.

**Figure 2:**
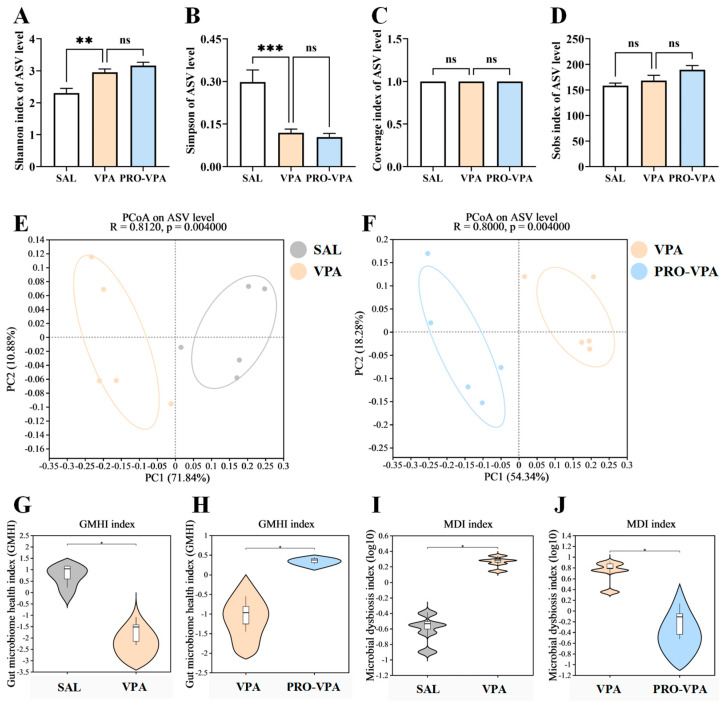
L-proline modulates the structure of gut microbiota in the mouse model for ASD. (**A**) Shannon index. (**B**) Simpson index. (**C**) Coverage index. (**D**) Sobs index. Statistically significant FDR correction *** *p* < 0.001, one-way ANOVA. (**E**) PCoA of the microbiota based on the weighted UniFrac distance metrics between the SAL and VPA groups. ANOSIM, R = 0.8120, *p* = 0.004. (**F**) PCoA of the microbiota based on the weighted UniFrac distance metrics between the VPA and PRO-VPA groups. ANOSIM, R = 0.8000, *p* = 0.004. (**G**) Difference analysis of GMHI between the SAL and VPA groups. (**H**) Difference analysis of GMHI between the SAL and VPA groups. (**I**) Difference analysis of MDI between the SAL and VPA groups. (**J**) Difference analysis of MDI between the SAL and VPA groups. Significance was set at ns, not significant (*p* > 0.05), * *p* < 0.05, ** *p* < 0.01, and *** *p* < 0.001. *n* = 5.

The α diversity indices were evaluated at the ASV level. Our findings revealed that community diversity was significantly elevated in the VPA group compared to the SAL control group, as indicated by a higher Shannon index and a lower Simpson index (Figure 2A,B). However, the VPA treatment did not significantly affect the community coverage (assessed by the Coverage index, Figure 2C) or the community richness (assessed by the Sobs index, Figure 2D) of the gut microbiota. Furthermore, L-proline supplementation did not exert a statistically significant effect on α diversity in the ASD mouse model (Figure 2A–D). PCoA was utilized to examine the community structures of the gut microbiota. The results demonstrated that the gut microbiota of mice in the VPA group was distinct from that of the SAL group (R = 0.8120, *p* = 0.004, Figure 2E). Moreover, the gut microbiota of mice in the PRO-VPA group was separated from that of the VPA group (R = 0.8000, *p* = 0.004, Figure 2F), suggesting that L-proline supplementation significantly modulated the gut microbial community structure in the VPA-treated mice.

The gut microbiome health index (GMHI) was employed as a metric to assess the health status of the microbiota [26]. Extending this observation, we further discovered that the GMHI was decreased in mice from the VPA group compared to those in the SAL group (Figure 2G). Notably, the L-proline treatment was found to significantly elevate the GMHI in the ASD mouse model (Figure 2H). Additionally, the microbial dysbiosis index (MDI) was calculated to quantify the degree of microbial imbalance. The results indicated a significant increase in MDI in the ASD mice compared to those in the SAL group (Figure 2I). Concurrently, L-proline supplementation significantly decreased the MDI in the VPA-induced ASD mouse model (Figure 2J). Collectively, these findings suggest that the L-proline treatment can markedly enhance the health status of the microbiota and ameliorate the degree of gut microbiota dysbiosis.

### 3.3. L-Proline Reshapes the Composition of Gut Microbiota in the VPA-Induced Mouse Model of ASD

**Figure 3:**
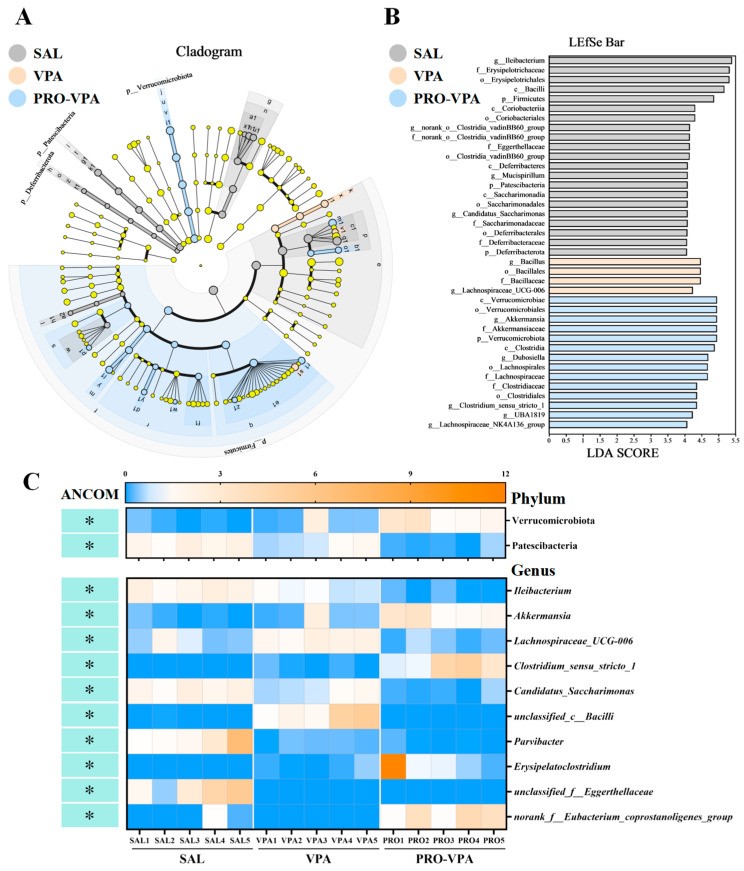
Identification of characteristic taxa with the most significant difference among the three experimental groups. (**A**) Cladograms generated by LEfSe indicate differences in the bacterial taxa among the SAL, VPA, and PRO-VPA groups. (**B**) LDA scores for the bacterial taxa are differentially abundant among the SAL, VPA, and PRO-VPA groups (LDA > 4). (**C**) A heatmap depicts the differential abundance of microbial taxa that varied among the SAL, VPA, and PRO-VPA groups. Rows (microbial taxa at the phylum and genus level) and columns (samples) were ordered by hierarchical clustering. Differentially abundant taxa were determined by ANCOM (W > 0.6) and labeled with * in the heatmap. *n* = 5.

LDA was integrated with LEfSe to identify the ASVs that were primarily responsible for the compositional differences observed in the gut microbial communities. A comprehensive analysis yielded a total of 38 bacterial clades, characterized by LDA > 4 and *p* < 0.05, which were utilized to elucidate the gut microbial variations across different treatment groups, spanning from the phylum to the genus level (Figure 3A,B). The dominant species in the SAL group were *Firmicutes*, *Patescibacteria*, and *Deferribacterota* at the phylum level; *Bacilli*, *Coriobacteriia*, *Deferribacteres*, and *Saccharimonadia* at the class level; *Erysipelotrichales*, *Coriobacteriales*, *Clostridia_vadinBB60_group*, *Saccharimonadales*, and *Deferribacterales* at the order level; *Erysipelotrichaceae*, *norank_o__Clostridia_vadinBB60_group*, *Eggerthellaceae*, *Saccharimonadaceae*, and *Deferribacteraceae* at the family level; and *norank_o__Clostridia_vadinBB60_group*, *Mucispirillum*, *Candidatus_Saccharimonas*, and *Ileibacterium* at the genus level. The dominant species in the VPA group were *Bacillales* at the order level; *Bacillaceae* at the family level; and *Bacillus* and *Lachnospiraceae_UCG-006* at the genus level. The dominant species in the PRO-VPA group were *Verrucomicrobiota* at the phylum level; *Clostridia* and *Verrucomicrobiae* at the class level; *Clostridiales*, *Lachnospirales*, and *Verrucomicrobiales* at the order level; *Akkermansiaceae*, *Clostridiaceae*, and *Lachnospiraceae* at the family level; and *Akkermansia*, *Dubosiella*, *Lachnospiraceae_NK4A136_group*, and *UBA1819* at the genus level.

Given the potential for high false-positive rates associated with LEfSe, the ANCOM method was employed as an additional tool to refine the identification of bacterial taxa enriched across group comparisons [19]. At a significance threshold of W > 0.6, the analysis revealed differential abundances of two phyla and ten genera of gut bacteria among the SAL, VPA, and PRO-VPA groups (Figure 3C). Notably, two phyla (*Patescibacteria* and *Verrucomicrobiota*) and four genera (*Ileibacterium*, *Candidatus_Saccharimonas*, *Lachnospiraceae_UCG-006*, and *Akkermansia*) were also identified as differentially abundant bacterial taxa in the LEfSe analysis (Figure 3A,B). These findings indicated that ANCOM refined the identification of enriched bacterial taxa, confirming that *Patescibacteria* at the phylum level and *Ileibacterium* and *Candidatus_Saccharimonas* at the genus level were differentially enriched in the SAL group; *Lachnospiraceae_UCG-006* at the genus level was enriched in the VPA group; and *Verrucomicrobiota* at the phylum level and *Akkermansia* at the genus level were enriched in the PRO-VPA group. Collectively, these results suggested that the L-proline treatment significantly altered the gut microbiome composition in the VPA-induced ASD mouse model (Figure 3C).

### 3.4. L-Proline Changes the Contents of Metabolites in the Mouse Cecum

**Figure 4:**
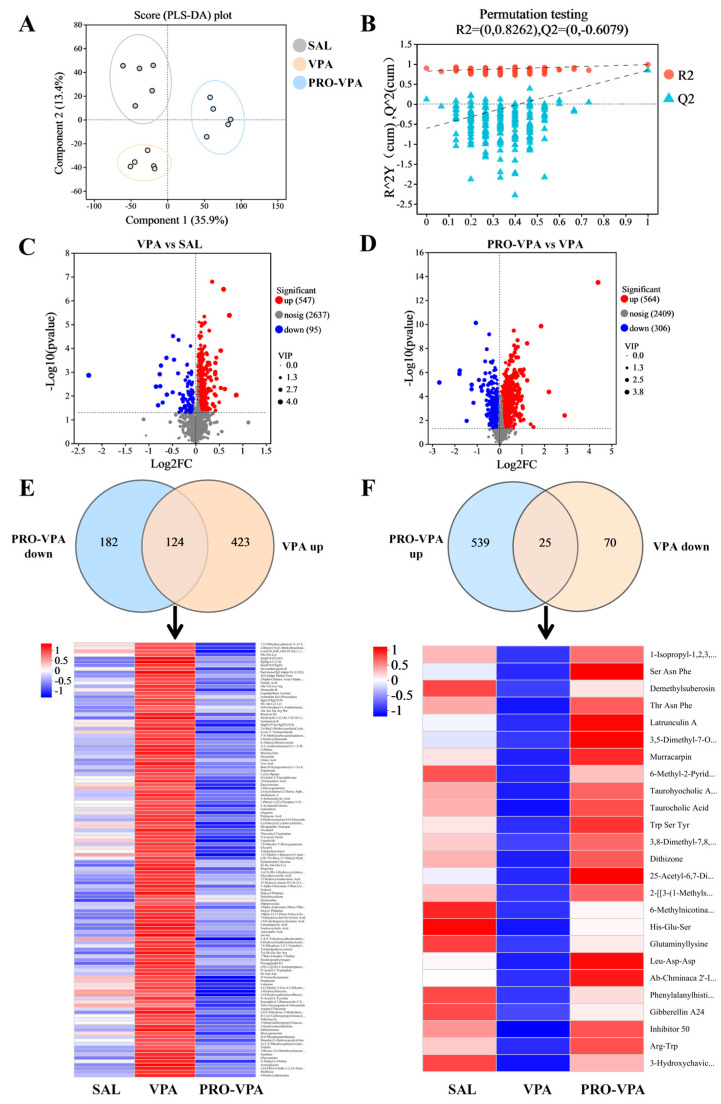
Metabolomic analysis of cecal contents reveals metabolic alterations in VPA-induced mouse model for ASD following the L-proline treatment. (**A**) Partial least squares discriminant analysis (PLS-DA). (**B**) Permutation test. (**C**) Volcano plots of the differential metabolites between the SAL and VPA groups. (**D**) Volcano plots of the differential metabolites between the VPA and PRO-VPA groups. (**E**) Venn diagram of up-regulated metabolites in VPA vs. SAL and down-regulated metabolites in PRO-VPA vs. VPA, as well as the heat map of their overlapped metabolites. (**F**) Venn diagram of down-regulated metabolites in VPA vs. SAL and up-regulated metabolites in PRO-VPA vs. VPA, as well as the heat map of their overlapped metabolites. *n* = 5.

The regulatory impacts of L-proline on microbiota-derived metabolites were examined through a metabolomics approach. PLS-DA revealed significant disparities in metabolite composition among the SAL, VPA, and PRO-VPA groups, suggesting unique gut metabolomic alterations in the ASD mice subsequent to L-proline administration (Figure 4A). The predictive accuracy of the PLS-DA model was confirmed by permutation test results (Figure 4B). Volcano plots were employed to elucidate the differential metabolites between the SAL and VPA groups, as well as between the PRO-VPA and VPA groups, thereby highlighting the modulated metabolites (Figure 4C,D). Specifically, compared to the SAL group, the VPA group exhibited 547 up-regulated and 95 down-regulated metabolites (Figure 4C). Following L-proline intervention, the PRO-VPA group demonstrated 564 up-regulated and 306 down-regulated metabolites (Figure 4D). Figure 4E illustrates the Venn diagram of up-regulated metabolites in the VPA vs. SAL comparison and down-regulated metabolites in the PRO-VPA vs. VPA comparison, accompanied by a heat map depicting their 124 overlapped metabolites. Similarly, Figure 4F displays the Venn diagram of down-regulated metabolites in the VPA vs. SAL comparison and up-regulated metabolites in the PRO-VPA vs. VPA comparison, along with a heat map of their 25 overlapped metabolites. These observations indicate that L-proline exerts a reversal effect on the regulatory influence of the VPA treatment on gut metabolites in mice, leading to a gut metabolic profile in the PRO-VPA group that more closely resembles that of the SAL group (Figure 4E,F).

### 3.5. Pathway Enrichment Analysis of Metabolic Pathways

**Figure 5:**
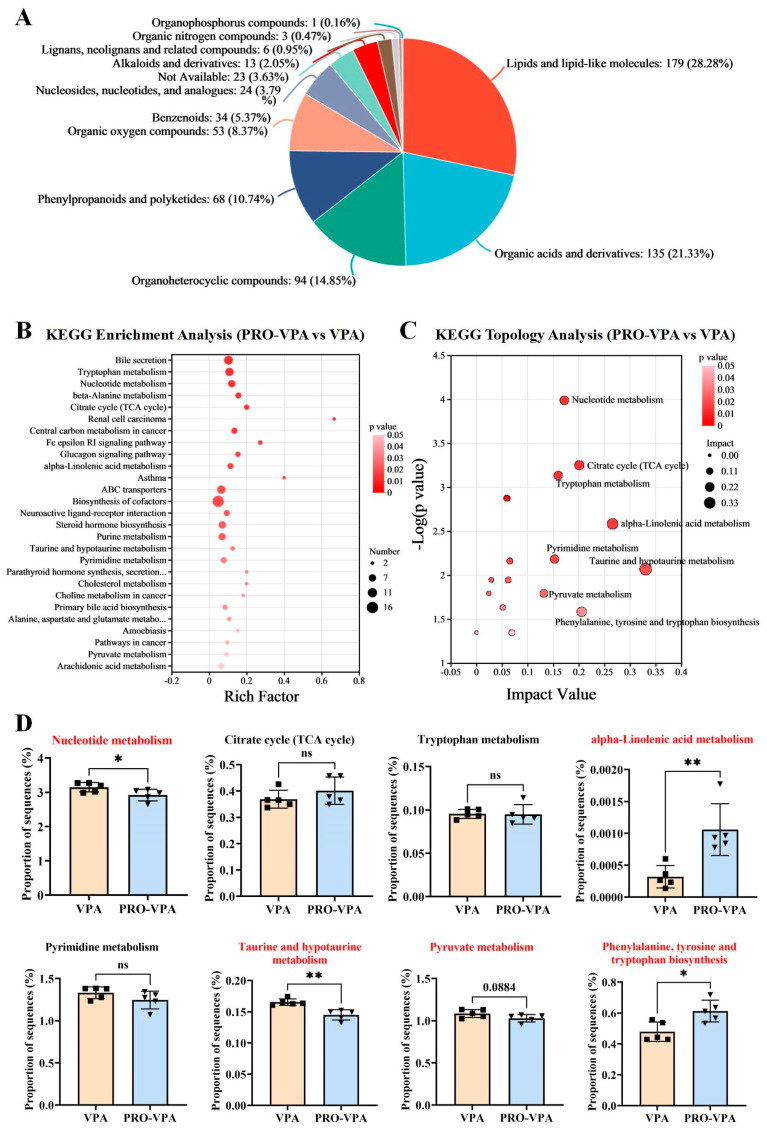
Classification and functional enrichment analysis of gut differential metabolites between the VPA and PRO-VPA group. (**A**) The proportion of differential gut metabolite categories. (**B**) Metabolic pathway enrichment of differential metabolites. (**C**) Topology analysis of metabolic pathways. (**D**) Metagenome prediction from PICRUSt in the ASD mice after the L-proline treatment. Nucleotide metabolism, taurine and hypotaurine metabolism, and pyruvate metabolism were decreased significantly, whereas alpha-linolenic acid metabolism and phenylalanine, tyrosine, and tryptophan biosynthesis were increased by the L-proline treatment in the ASD mice. The bars indicate mean ± SEM values. The data were statistically analyzed using our unpaired two-tailed Student’s *t*-test. Significance was set at ns, not significant (*p* > 0.05), * *p* < 0.05 and ** *p* < 0.01. *n* = 5.

To gain a deeper understanding of the molecular mechanisms through which L-proline alleviates ASD-like behavioral abnormalities, this study primarily concentrated on the 870 differential metabolites identified between the PRO-VPA and VPA groups. Metabolite annotation was conducted using the HMDB, revealing that 237 metabolites could not be annotated at the Superclass level. The remaining 633 metabolites were categorized as follows: lipids and lipid-like molecules (28.28%), organic acids and derivatives (21.33%), organoheterocyclic compounds (14.85%), phenylpropanoids and polyketides (10.74%), organic oxygen compounds (8.37%), benzenoids (5.37%), nucleosides, nucleotides, and analogues (3.79%), alkaloids and derivatives (2.05%), lignans, neolignans, and related compounds (0.95%), organic nitrogen compounds (0.47%), organophosphorus compounds (0.16%), and an additional 3.63% that were not classifiable within these categories (Figure 5A).

A rigorous exploration into the significance of these metabolites was conducted through KEGG pathway enrichment analysis. An analysis of the 870 differential metabolites between the PRO-VPA and VPA groups using KEGG enrichment revealed that L-proline exerted a significant influence on 27 pathways in the VPA-treated mice (Figure 5B, *p* < 0.05). Subsequently, to gain further insights into the biological pathways involved in the metabolism of the differentially expressed metabolites and their functional roles, pathway enrichment and topology analyses were performed using MetaboAnalyst (Version 5.0). Based on the identified metabolites and their concentration changes, eight metabolic pathways emerged with a pathway impact value > 0.1, which served as the threshold for relevance. These eight metabolic pathways, which were significantly regulated by L-proline in mice exhibiting ASD-like characteristics, encompassed nucleotide metabolism, citrate cycle (TCA cycle), tryptophan metabolism, alpha-linolenic acid metabolism, pyrimidine metabolism, taurine and hypotaurine metabolism, pyruvate metabolism, and phenylalanine, tyrosine, and tryptophan biosynthesis (Figure 5C, *p* < 0.05 and impact value > 0.1). The differential metabolites between the VPA and PRO-VPA groups involved in the eight metabolic pathways are presented in Table 1.

Furthermore, the findings from the metabolomic analysis were substantiated and corroborated by the predicted functional analysis of the gut microbiota. To gain insight into how microbial alterations induced by L-proline supplementation influenced mouse metabolism, PICRUSt was utilized to predict the potential functional pathways that could be affected by the gut microbiota. As anticipated from the microbiome data, the L-proline treatment in mice exhibiting ASD-like characteristics resulted in a significant decrease in the pathways of nucleotide metabolism, taurine and hypotaurine metabolism, and pyruvate metabolism, while it increased the pathways of alpha-linolenic acid metabolism and phenylalanine, tyrosine, and tryptophan biosynthesis (Figure 5D). Additionally, the pyruvate metabolic pathway exhibited a tendency to be reduced in the PRO-VPA group compared to the VPA group, albeit with a marginal significance (*p* = 0.0884). Collectively, these results demonstrated that the metabolomic analyses aligned with several key observations from the microbiome studies, offering valuable insights into the preventive effects of L-proline on ASD-like behavioral disorders.

### 3.6. Correlation Analysis of Gut Microbiota and Metabolites

**Figure 6:**
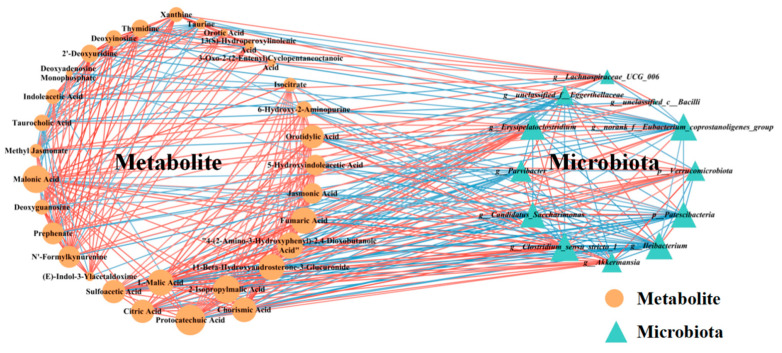
Correlation network analysis between the 31 differential metabolites involved in eight metabolic pathways significantly regulated by L-proline (as shown in Table 1) and the differentially enriched gut bacteria (including two phyla and ten genera, identified by ANCOM). Differential metabolites (depicted as orange circles) and gut microbiota (represented as blue triangles) are visualized as nodes. The size of each node is indicative of its degree, reflecting the number of connections to other nodes within the network. The lines connecting these nodes represent statistically significant Spearman correlation coefficients (with *p* < 0.05 and |coefficient| > 0.6). Specifically, red links signify positive interactions between nodes, whereas blue links denote negative interactions.

To gain a deeper understanding of the relationship between the drastically altered gut microbiota and gut metabolites, we conducted an analysis to identify correlations between the 31 differential metabolites involved in eight metabolic pathways significantly regulated by L-proline (as shown in Table 1), and the differentially enriched gut bacteria (comprising two phyla and ten genera, as determined by ANCOM). This analysis was performed using the Spearman correlation method. In constructing the correlation network, only strong (|coefficient| > 0.6) and statistically significant (*p* < 0.05) correlations were considered for inclusion (Figure 6). The findings indicate that the network comprises 394 statistically significant correlations among the 31 differential metabolites and 12 differential gut bacteria (Figure 6), thereby providing a visual depiction of the intricate interdependencies between the gut microbiota and metabolites.

Several central nodes emerged in the network, forming numerous connections and, thus, likely serving as hubs of physiologically significant interactions. Based on the node degree, the top the gut microbiota identified were *Clostridium_sensu_stricto_1*, *Ileibacterium*, and *norank_f__Eubacterium_coprostanoligenes_group* in descending order (Figure 6). Notably, the genus *Clostridium_sensu_stricto_1* exhibited extensive connectivity within the network, demonstrating correlations with 25 nodes included in the correlation analysis. Furthermore, *Ileibacterium* and *norank_f__Eubacterium_coprostanoligenes_group* displayed significant and strong correlations with 24 nodes, respectively (Figure 6). Moreover, according to the node degree from high to low, protocatechuic acid, 2-isopropylmalic acid, and malonic acid represented the top three differential metabolites, showing significantly strong correlations with 25, 24, and 24 nodes, respectively (Figure 6). These findings suggest robust associations between the gut microbiota and gut metabolites influenced by L-proline. Collectively, these results imply that L-proline’s capacity to prevent autism-like behaviors may be linked to its regulatory effects on the interactions and dynamic balances between gut microbial composition and gut metabolites.

## 4. Discussion

The primary objective of the present research was to assess the potential preventive effects of L-proline supplementation on autistic-like symptoms in a mouse model of ASD induced by VPA. Our underlying hypothesis posited that supplementing the diet of offspring exposed to VPA in utero would alter the composition of the gut microbiota and the profiles of gut metabolites, ultimately leading to enhanced health outcomes. The findings revealed that L-proline supplementation significantly prevented ASD-like behaviors in offspring exposed to VPA in utero. This mitigation was accompanied by the restoration of key bacterial taxa and modifications in the profiles of gut metabolites. These results imply the potential of L-proline as a nutritional intervention for preventing behavioral symptoms associated with ASD.

In our study, which focused on microbial analysis, we examined the impact of L-proline supplementation on the gut microbial composition of mice exposed to VPA during pregnancy. Our findings revealed that L-proline had a notable influence on the community and structure of the gut microbiome. Although L-proline supplementation did not affect α diversity in the ASD mouse model with any statistical significance, aligning with our previous research [16], it markedly decreased the GMHI, which differentiates healthy from non-healthy groups more effectively than commonly utilized ecological indices, such as Shannon diversity and richness, which are generally regarded as indicators of gut health and dysbiosis [26]. Taken together, these results imply that the L-proline treatment can substantially ameliorate the severity of gut microbiota dysbiosis.

Notably, our ANCOM and LEfSe analyses of the gut microbiota collectively indicate that L-proline supplementation enhances the abundance of *Verrucomicrobia* at the phylum level and *Akkermansia* at the genus level (Figure 3). L-Proline is a versatile non-essential amino acid that plays pivotal roles in oxidative stress protection, protein synthesis, and energy metabolism [16,27]. Faure et al. demonstrated that dietary supplementation with L-proline can enhance mucin synthesis [28], while *Akkermansia muciniphila* is an anaerobic bacterium that specializes in the degradation of mucin, efficiently utilizing mucus as its primary carbon and nitrogen source [29,30]. These results provide a possible hypothesis that L-proline increases the abundance of *Akkermansia*: L-proline may serve as an energy and nutrient source for *Akkermansia* by stimulating mucin synthesis, potentially leading to an increased abundance of this genus. However, further experimental validation is required to substantiate this hypothesis.

Recent research has highlighted the remarkable potential of *Akkermansia muciniphila* in promoting intestinal health, albeit with limited investigation into its role in neurodevelopment [31]. The literature provides some support for a decrease in the abundance of the mucolytic bacterium *Akkermansia muciniphila*, a member of the *Verrucomicrobia* phylum, in the feces of children with autism [32,33]. In a study of infants later diagnosed with ASD from the All Babies in Southeast Sweden cohort, *Akkermansia muciniphila* was absent and inversely correlated with gastrointestinal and mood symptoms in early childhood, suggesting a robust connection between disruptions in mucin health and ASD [34]. *Akkermansia muciniphila* plays a crucial role in maintaining the gut barrier, alleviating gut inflammation, and regulating metabolites to modulate the central nervous system [35,36]. This bacterium facilitates mucin degradation and produces folate, propionate, and acetate; it is renowned for enhancing enterocyte monolayer integrity, fortifying compromised gut barriers, and possessing immunomodulatory properties [34]. The aforementioned findings suggest that the administration of L-proline as a supplemental intervention for the prevention of ASD symptoms may be linked to an augmentation in the abundance of the beneficial bacterium *Akkermansia* in mouse models of ASD.

Furthermore, the ANCOM and LEfSe analyses have collectively demonstrated that L-proline is capable of reducing the abundance of *Candidatus_Saccharimonas*, a genus previously identified as characteristic of offspring exposed to VPA [37,38]. Notably, this genus exhibits a positive correlation with time spent in an empty chamber and self-grooming behavior, while demonstrating a negative correlation with time spent in a chamber containing a stranger rat and social sniffing time [37]. In addition, various reports indicate that the abundance of *Candidatus_Saccharimonas* is elevated in individuals suffering from conditions such as colitis [39,40], lupus [41], and glycolipid metabolic dysfunction [42], showing a potentially detrimental impact on immune function [43]. Overall, these findings hint at the pleiotropic role of L-proline in preventing autism symptoms by modulating the gut microbiota through a complex network matrix. However, it is imperative that further in-depth studies be conducted to definitively elucidate the underlying mechanisms of their efficacy.

Gut bacteria engage in interactions with the brain via neural, immune, and metabolite pathways [44]. Additionally, alterations in the microbiome frequently lead to modifications in metabolic profiles, which in turn influence the availability and diversity of nutrients and microbial metabolites [15]. Microorganisms produce metabolites and neurotransmitters, serving as chemical signals capable of traversing the blood–brain barrier. These signals can either directly or indirectly impact the vagus nerve, thereby modulating the central nervous system [45,46]. Indeed, metabolomic analyses of serum, feces, and urine samples from individuals with ASD have revealed disparities in various molecules compared to typically developing individuals, with numerous dysregulated compounds traced back to microbial origins [45,47,48,49,50]. In the present study, the findings demonstrated that L-proline supplementation regulates the gut metabolic profile of the ASD mice (Figure 4) and effectively mitigates ASD-like behavioral disorders. The predicted functional analysis of the gut microbiota, coupled with KEGG pathway enrichment analysis of metabolomics, corroborated that L-proline attenuates pathways implicated in nucleotide metabolism, taurine and hypotaurine metabolism, and pyruvate metabolism. Concurrently, it enhances pathways associated with alpha-linolenic acid metabolism and phenylalanine, tyrosine, and tryptophan biosynthesis (Figure 5).

Among the various metabolic pathways examined, taurine and hypotaurine metabolism has been notably identified as the most disrupted in both plasma and urine samples of patients with ASD [51]. In the present study, L-proline supplementation significantly elevated the levels of taurocholic acid, sulfoacetic acid, and taurine, which are key components of the taurine and hypotaurine metabolism pathways (Table 1). Consistent with our findings, Liu et al. demonstrated that dietary supplementation with L-proline resulted in elevated concentrations of taurine in both plasma and amniotic fluid [52]. This observation provides support for the hypothesis that one potential mechanism of action for L-proline involves increasing taurine levels in the circulation and brain. This area will serve as a pivotal focus of our future research endeavors.

Taurine, one of the most abundant amino acids in the brain, functions as a neuromodulator, regulating the balance between excitatory and inhibitory neuronal activity [53]. Furthermore, taurine has been proposed to exhibit multiple positive effects, including antioxidant, anti-inflammatory, gut-regulatory, and immune-modulatory properties, which may alleviate some symptoms associated with ASD [15,54,55]. Currently, there is a consensus in the literature that taurine plays a protective role in ASD [51]. The aforementioned findings suggest that the preventive effect of L-proline on ASD-like behavioral disorders may be attributed to its modulation of the taurine and hypotaurine metabolism pathway and the subsequent increase in taurine levels in the ASD mice.

Despite the insights provided by our study, several limitations merit attention. Firstly, the sample size utilized in our investigation is relatively limited, which may restrict the generalizability of our findings. To facilitate more in-depth exploration and enhance the robustness of our conclusions, future research studies should endeavor to expand the sample size. Secondly, our analysis was confined exclusively to male mice, thereby neglecting potential sex-specific differences. To ensure a comprehensive understanding of the phenomenon under investigation, future studies should incorporate female mice as well. Based on the clinical literature research, multiple studies have indicated a significant male predominance in the ASD population, with ratios of males to females exceeding 2:1 and even reaching 5:1 [1,56,57]. Therefore, we have prioritized the use of male animals in our experiment. However, approximately 20% of the human populations surveyed were female. We acknowledge the limitations of conducting studies only on one sex, and a study of sex differences in the effect of L-proline supplementation on autism-like behavior would also be of great interest for our future studies. Lastly, the interplay between gut microbiota and various metabolites is intricate and influenced by a multitude of factors. Consequently, we acknowledge that it was not feasible to exclude all potential confounding variables in our study. This underscores the need for the meticulous and rigorous control of confounding factors in subsequent research endeavors to further elucidate the underlying mechanisms and relationships.

L-Proline is rich in different types of foods, such as cheese and soy (3–4 g L-proline/100 g), and has gained widespread utilization as a component in supplements, health foods, and cosmetics [58]. According to the formulation proposed by Reagan-Shaw et al.: human equivalent dose = animal dose  ×  human K_m_/animal K_m_ [59], the dose of L-proline fed to mice (1%, 1000 mg/kg) in this experiment is equal to 4.86 g/day for a 60 kg adult and 2.4 g/day for a 20 kg child. Moreover, the no-observed-adverse-effect-level for L-proline was determined to be a dietary dose of 5.0% (2772.9 mg/kg body weight/day for males and 3009.3 mg/kg body weight/day for females) in a 90-day feeding study with Fischer 344 rats [58]. Additionally, a clinical study was conducted to investigate the effects of prolonged oral administration of L-proline. This study involved four patients, who were administered supplementary L-proline at dosages of 2 to 10 g per day for a duration spanning 2 to 5 years and no significant adverse effects were observed throughout the study period [60]. These above studies suggest that the dose of L-proline used in our study was also designed to be realistic, reasonable, and safe.

We did not observe any indications of problems caused by the administration of L-proline during pregnancy. In addition, studies have demonstrated that L-proline plays several roles in the development of the placenta, conceptus, and fetus [61,62], and dietary L-proline supplementation during gestation enhances fetal survival and placental development in mice [52,63]. Our current study serves as a preliminary exploration of the potential of L-proline in mitigating ASD-like behavioral impairments in mice. Additional studies are warranted to elucidate the effects of L-proline on ASD and to accumulate the necessary evidence before considering the clinical application of this compound for the prevention and treatment of ASD.

Researchers are working hard to elucidate the etiology of ASD and trying to find potential gut microbiota as candidate biomarkers for ASD diagnosis [64,65]. Despite some recognizable patterns that have been obtained, most microorganisms from phylum to species have different results in different studies [66,67]. Thus, to date, gut microbial composition alone does not provide predictive biomarkers for ASD, and further studies are needed to reach a consensus. High-throughput sequencing needs to be combined with omics data from multiple sources, such as proteomics, transcriptomics, metabolomics, microRNAs, and exosomes, to generate potential signatures for the symptom spectrum of individuals with ASD [64].

## 5. Conclusions

This study elucidates the potential efficacy of dietary L-proline in preventing autistic behaviors, as demonstrated by a reduction in repetitive behaviors and an amelioration of social impairments observed in a VPA-induced ASD mouse model. Furthermore, L-proline modulates the composition and structure of gut microbiota. The ANCOM and LEfSe analyses of gut microbiota collectively indicate that L-proline supplementation enhances the abundance of the *Verrucomicrobia* at the phylum level and the *Akkermansia* at the genus level, while concurrently reducing the abundance of the *Patescibacteria* at the phylum level, as well as the *Ileibacterium*, *Candidatus_Saccharimonas*, and *Lachnospiraceae_UCG-006* at the genus level in the VPA-induced mouse model for ASD. Additionally, L-proline also alters the profiles of gut metabolites. Functional analysis of the gut microbiota and KEGG pathway enrichment analysis of differential metabolites between the PRO-VPA and VPA groups corroborate that L-proline attenuates pathways involved in nucleotide metabolism, taurine and hypotaurine metabolism, and pyruvate metabolism, while enhancing those related to nucleotide metabolism, taurine and hypotaurine metabolism, and pyruvate metabolism. In summary, the regulatory effects of L-proline on gut microbiota and its metabolites may constitute a pivotal mechanism underlying its prevention of autism-like behavior disorder. These findings propose that dietary L-proline may offer novel intervention strategies for the prevention of autistic symptoms.

## Figures and Tables

**Table 1:** The eight metabolic pathways and differential metabolites regulated by L-proline in the mouse model of ASD, with impact value > 0.1 and *p* < 0.05.

Pathway Description	Hits	Total	Impact Value	*p* Value	Metabolite	Regulate (PRO-VPA vs. VPA)
Nucleotide metabolism	7	56	0.17123	0.00010	deoxyadenosine monophosphate	down
					6-hydroxy-2-aminopurine	up
					2′-deoxyuridine	down
					deoxyinosine	down
					thymidine	down
					deoxyguanosine	down
					xanthine	down
Citrate cycle (TCA cycle)	4	20	0.20060	0.00056	fumaric acid	up
					citric acid	up
					L-malic acid	up
					isocitrate	up
Tryptophan metabolism	6	56	0.15937	0.00073	5-hydroxyindoleacetic acid	up
					indoleacetic acid	down
					(E)-indol-3-ylacetaldoxime	down
					4-(2-amino-3-hydroxyphenyl)-2,4-dioxobutanoic acid	up
					11-beta-hydroxyandrosterone-3-glucuronide	up
					*N*′-formylkynurenine	down
alpha-Linolenic acid metabolism	4	30	0.26546	0.00260	jasmonic acid	up
					methyl jasmonate	down
					3-oxo-2-(2-entenyl)cyclopentaneoctanoic acid	down
					13(S)-hydroperoxylinolenic acid	down
Pyrimidine metabolism	5	62	0.15253	0.00654	orotic acid	down
					2′-deoxyuridine	down
					thymidine	down
					orotidylic acid	up
					malonic acid	up
Taurine and hypotaurine metabolism	3	22	0.33015	0.00851	taurocholic acid	up
					sulfoacetic acid	up
					taurine	up
Pyruvate metabolism	3	28	0.13147	0.01606	fumaric acid	up
					2-isopropylmalic acid	up
					L-malic acid	up
Phenylalanine, tyrosine, and tryptophan biosynthesis	3	34	0.20534	0.02603	chorismic acid	up
					prephenate	down
					protocatechuic acid	up

## Data Availability

Data supporting reported results can be requested after publication from the corresponding author.
